# Construction of an AI-Assisted Pedigree Mapping System for Hereditary Gynecological Tumors: Algorithm Development and Pilot Validation Study

**DOI:** 10.2196/78080

**Published:** 2026-10-15

**Authors:** Jing Xu, Yajun Wang, Hui Wang, Jiayang Sun, Xingang Lei, Congjian Xu, Yu Kang

**Affiliations:** 1 Obstetrics and Gynecology Hospital of Fudan University, Fangxie Road, No. 419, Shanghai, China, China, 86 33189900; 2 Shanghai Taihealth Intelligent Technology Co, Ltd, Shanghai, China, China; 3 Westlake University, Hangzhou, China; 4 Department of Gynecology, Affiliated Hangzhou First People's Hospital, School of Medicine, Westlake University, Hangzhou, China

**Keywords:** hereditary gynecological tumors, artificial intelligence, AI, pedigree mapping, retrieval-augmented generation, large language models

## Abstract

**Background:**

Pedigree mapping serves as the first step in genetic counseling. Traditional methods relying on manual pedigree drawing and information collection are inefficient and prone to errors.

**Objective:**

This study aimed to design an intelligent pedigree mapping system based on a specialized model, intended to automate family history collection and standardized pedigree map generation through conversational interaction, thereby enhancing the clinical efficiency of genetic counseling.

**Methods:**

This study adopted a participatory design and iterative development approach. We formed an interdisciplinary team comprising gynecologic oncologists, genetic counselors, AI engineers, and patient representatives. The system was built around a proprietary, specialized large language model for gynecologic oncology, featuring a microservices architecture that included pedigree data collection. Through 4 iterative cycles, we integrated specialized knowledge bases using retrieval-augmented generation technology and applied a novel graph-based kinship inference and validation module. The system’s performance was comprehensively evaluated based on the accuracy of pedigree map generation and user feedback on usability.

**Results:**

We significantly improved system performance by optimizing dialogue strategies, expanding the knowledge base, and applying self-developed algorithms to improve data extraction accuracy. The initial database of the system defined 100 core information nodes and matched approximately 800 user expression examples, while the final pilot version of the database was expanded to 245 core information nodes and 2256 user expression examples. Although the AI function was limited in the early stage, the final version of the system demonstrated outstanding accuracy in generating genealogy nodes and relationships. Test users generally gave positive reviews of the system’s innovation, ease of use, and intuitiveness of information presentation, believing it to have great potential in clinical practice.

**Conclusions:**

The developed system is an accurate, efficient auxiliary tool, and pilot validation showed promising preliminary performance. After pilot clinical validation, it holds promise for integration into routine clinical workflows to optimize genetic counseling services.

## Introduction

Hereditary gynecological tumors account for up to 15% to 20% of all gynecological malignancies [1,2]. Genetic counseling [3,4] is crucial for early screening, intervention, and personalized treatment decisions for high-risk individuals and is a critical step in reducing the risk of hereditary tumors and improving treatment outcomes. The traditional model of genetic counseling is face-to-face, but remote consultation, including telephone consultation, video consultation, and online consultation, has gradually become an integral part of genetic counseling in recent years [5].

Pedigree mapping serves as the first step in genetic counseling. However, current clinical practice faces severe challenges: the genetic counseling process heavily relies on manual family history collection and pedigree drawing by physicians or counselors. When dealing with families involving multiple generations and complex kinship relationships (eg, half-siblings and adoption), the rates of information omission and error increase significantly. This not only consumes valuable consultation time but also severely limits the accessibility and standardization of genetic counseling services.

In recent years, the rapid advancement of AI, particularly large language models (LLMs), has brought revolutionary opportunities to address this clinical bottleneck [6,7]. While general-purpose LLMs demonstrate powerful language understanding capabilities [8], their tendency toward clinical hallucination—generating inaccurate or unsubstantiated medical information—poses a serious safety risk in the highly specialized medical field. Therefore, developing specialty-specific large models with medical knowledge has become an imperative trend [9].

Against this backdrop, this study aimed to design, develop, and validate a pilot version of an intelligent auxiliary pedigree mapping system based on a gynecological oncology specialty LLM. To ensure the clinical practicality and user acceptance of the system, this study adopted a participatory design approach throughout the entire process. Senior gynecologic oncologists, genetic counselors, and patient representatives were involved in all stages, ensuring that the final product could be seamlessly integrated into existing clinical workflows and effectively address users’ core points. The system achieved the key functions through preconsultation multiturn dialogue with users: automatically and accurately extracting structured personal and family history information and generating standardized, visual pedigree charts in real time.

This study’s core objectives were to design an intelligent pedigree mapping system to enhance the clinical efficiency of genetic counseling and provide a replicable and scalable “China-Shanghai Solution” for deeply empowering clinical precision medicine with AI technology.

## Methods

### Design Population and Patient Engagement

To ensure the clinical practicality and national applicability of the system, we invited senior gynecologic oncologists, genetic counselors, clinical research nurses, and patient representatives to participate in several online and offline seminars.

### System Design and Architecture

This study was a technological innovation and validation research that used participatory design and agile iterative development methods (Figure 1). The research process strictly adhered to ethical guidelines, with all involved patient data anonymized and the study approved by the relevant ethics committee.

**Figure 1.**
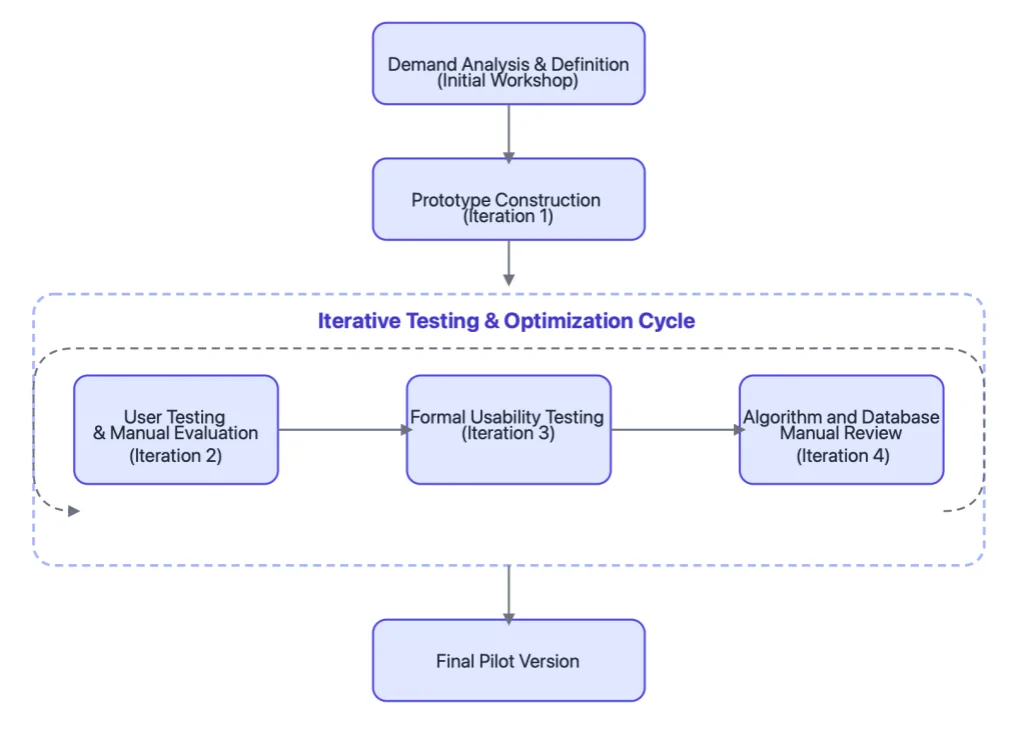
System design and development process.

### Build a Preliminary Dialogue and Genealogy Chart Generation Program

As the first step in building the system, multiple experienced genetic counselors collaborated with AI engineers to develop initial scripts for the system’s knowledge base and dialogue flows. To ensure the professionalism and accuracy of medical information, every guided question was reviewed by clinical genetics experts. Meanwhile, medical education specialists also participated to ensure that the dialogue language remained accessible and avoided potential misunderstandings caused by technical jargon.

When a user (patient) mentioned disease information about relatives (eg, “my mother’s sister was diagnosed with ovarian cancer at age 50”) during dialogue with the agent, the system’s back end natural language processing engine and specialized LLM worked collaboratively to perform entity recognition (identifying kinship relationships, diseases, and ages) and relationship extraction. Subsequently, using proprietary optimization algorithms, the system further enhanced the accuracy of relationship entities, automatically constructed a visualized pedigree chart according to genetic standards, and rendered it in real time.

### User Interface and System Architecture

We developed a mobile app that served as a user interface for both physicians and patients. The entire system was based on a microservices architecture, which ensured secure and efficient communication between the physician and the patient, as well as between the front end and back end natural language processors. To protect user privacy, all sensitive information was encrypted before data transmission, and conversation content was processed and stored anonymously, accessible only to authorized administrators for system optimization.

The system consisted of a base large model, 2 core agents, 3 support platforms, and 2 types of terminal apps. The base model was the self-developed multimodal medical model. The core agents were the family acquisition agent and the risk computation agent. The system provided services through 2 entrances: the physician side (web) and the patient side (app).

### Design and Test

#### Initial Workshop

We organized 6 symposiums nationwide, with the participation of 58 experts in the fields of gynecology, genetics, and information technology from different hospitals. These workshops provided us with a clear scope of the system’s core capabilities, defined initial conversations, and allowed us to collect a large amount of real-world data for dialogue database expansion.

#### User Testing

We invited 11 testers, including clinical geneticists, genetic counselors, research nurses, and 2 BRCA mutation carrier representatives, to test the prototype for 4 weeks. They were asked to have at least 5 independent conversations with the system during this time, each lasting at least 5 minutes, and to record the problems they encountered and their experience.

#### Formal Usability Testing

We recruited an additional 9 testers (7 health care workers and 2 patient representatives) to complete a series of preset tasks (eg, complete family history information input and interpret the system-generated family tree and risk warnings) in an observational environment. Project personnel recorded their operational paths and difficulties and collected feedback in real time.

### Datasets and Evaluation Procedure

The iterative process was initially planned to include 3 iterations, but during the development process, to address the accuracy of AI models when dealing with complex kinships, we decided to add a fourth iteration dedicated to manually reviewing and optimizing the genealogy map generation algorithm. Each iteration signified a significant expansion of the database and substantial improvements in system functionality.

We manually evaluated all conversations generated during the user testing and the generated family charts to assess system performance, categorizing the results into 3 categories: correct (accurate information extraction and correct family chart structure), wrong (missing key information or mismatched relationships), or fallback (the system could not understand the user’s answer and asked the user to phrase it in a different way).

All data used for tool iteration were derived from real-world, multiturn dialogue records generated by users during system use. At the initial stage of tool development, multiple experienced genetic counselors, clinical geneticists, and medical education specialists jointly reviewed the dialogue flow scripts, based on which the development team finalized requirements and established preliminary process rules. During iteration, the rules were continuously refined using real-world conversation data validated by experts; by contrast, dialogue data collected at user testing checkpoints served as an independent test set, and user feedback was systematically incorporated into the rule revision loop to ensure the objectivity and clinical relevance of improvements. The gold standard was jointly established by a panel of industry experts. Annotation, including entity recognition and attribute labeling of pedigree data, was primarily performed by relevant project personnel and 1 clinical staff member. All annotation disagreements were resolved through multiple offline discussions until consensus was reached, and the entire annotation process was strictly independent of the iterative optimization data to guarantee the reliability of the evaluation results.

### AI System Architecture

#### Specialized Medical LLM (Proprietary Medical LLM)

The core generative capability of this model was provided by an open-source model with a large parameter count, and no downstream task fine-tuning was performed on the model to maximally preserve the generalizability of the foundation model while reducing adaptation costs.

The overall dialogue workflow of the model was designed entirely based on the retrieval-augmented generation (RAG) architecture: during medical knowledge consultation dialogues, the agent retrieved highly relevant knowledge snippets from an external medical knowledge base according to the current dialogue context and injected them as augmented context into the model, thereby dynamically supplementing the model’s knowledge reservoir and compensating for the deficiencies of general-purpose foundation models in specialized medical domains.

Rule-based algorithms were used to constrain and calibrate the dialogue flow: through predefined step state identifiers and logical branches, the flow direction was strictly enforced to ensure that the interaction path conformed to the standard pedigree information collection protocol. These algorithms also performed real-time validation and constrained the model-generated responses, effectively suppressing factual errors and fabrications, thus substantially reducing model hallucination. By combining the language capabilities of the large model, external knowledge retrieval, and explicit rule-based control, the system achieved more controllable and trustworthy intelligent interaction in medical dialogue scenarios without requiring fine-tuning.

#### Patient-Side Data Collection

On the patient side, information was sequentially provided through dialogue, including gender, age, personal tumor history, family tumor history, kinship structure, age of onset, death status, and genetic testing information. The agent transformed unstructured statements into attribute-structured family history data through intent recognition, entity recognition, and kinship relation parsing, forming a standardized structure directly usable for pedigree chart rendering. To enhance extraction reliability, the agent cross-validated intermediate results by integrating risk rules, RAG retrieval outcomes, a rule engine, and structural consistency verification and automatically initiated follow-up questions for any uncertain information.

#### Final Output

On the basis of the extracted structured family history, the agent generated a pedigree chart and simultaneously performed risk assessment for hereditary breast and ovarian cancer syndrome, Lynch syndrome, and Peutz-Jeghers syndrome. Ultimately, the agent output genetic counseling recommendations.

### Safety and Hallucination Control

The agent was exclusively designed for gynecological oncology medical knowledge consultation, family history collection, and pedigree chart generation and did not output diagnostic or therapeutic decisions. The RAG system was restricted to a defined knowledge source, and all results were accompanied by source attribution. Knowledge extraction incorporated confidence filtering, rule-based validation, and human review; newly added knowledge must not conflict with existing high-confidence knowledge. Both the generated pedigree chart and the rules participated in result verification. These measures collectively supported the claim that the agent was subject to multilayered constraints and did not engage in free-form generation.

In the process of extracting family tumor history information and generating the pedigree chart, a mechanism of relationship confidence control and kinship consistency verification was introduced. For recognition results with low confidence or conflicting kinship relations, the agent did not directly incorporate them into the pedigree chart but instead triggered targeted follow-up questions. If the uncertainty persisted, a conservative fallback strategy was adopted, marking the relevant nodes or relationships as “unknown” or “pending confirmation.”

The generated pedigree chart was further validated against predefined kinship rules, including branch affiliation (paternal vs maternal), generational level, intragenerational and intergenerational relationships, key connecting kinships, and consistency checks for duplicate members. This approach thereby enhanced the logical consistency and traceability of the structured family tumor history results.

### Ethical Consideration

The present study involving clinical hereditary cancer information from patients was approved by the ethics committee of the Obstetrics and Gynecology Hospital of Fudan University (2021‐09-X1). Prior informed consent was obtained from all users, who were required to agree to relevant protocols and policies before participation; all collected data were deidentified prior to use to ensure that no personally identifiable information was involved.

## Results

In the first iteration phase, we built a systematic database of knowledge and dialogue based on the results of the workshop (Table 1). It defined 100 core information nodes (eg, specific kinship or disease) and matched them with approximately 800 recognizable user representation paradigms (recognition breadth ratio of 8.0). Informal testing of prototypes showed that 40% (40/100) of core information nodes had errors in genealogy generation, with the main problem being the inability to accurately identify complex or vague kinship titles such as “cousin.”

**Table 1.** The 4 iterative phases of system design and development.

	Phase			
	Iteration 1	Iteration 2	Iteration 3	Iteration 4
Method	Workshops and prototyping	User testing and manual evaluation	Formal usability test	Manual review of algorithms and databases
Participants, n	58	9	9	3 (core team)
Revision level	Preliminary construction	Major revisions	Major revisions	Major revisions
Fidelity level	Low	Low	Medium	High
Core information	Core requirements were clarified, but AI performance was insufficient	Key technical bottlenecks identified	Optimized user interface and interaction logic	The genealogy chart generation error rate was significantly reduced

Subsequent iteration step 2 (user testing) recorded a total of 822 user inputs (Table 2). We found that 519 (63.1%) inputs were processed efficiently, while 303 (36.9%) inputs triggered fallbacks because the system could not understand them. Of these, 69.2% (359/519) efficiently processed inputs were generated with correct family plot nodes, while a significant portion contained errors.

This observation prompted us to focus on reconstructing the identification logic of all kinship relationships. This iterative step eventually resulted in 50 new conversation scenarios, and the database expanded to 150 core information nodes and 1250 user expression examples.

**Table 2.** Overview of user testing in user interface and system architecture.

Characteristic	Value
Testers, n	9
Conversations recorded, n	71
Total test duration (min)	507
Average duration per conversation (minutes:seconds)	7:11
Total user input, n	822
Input that triggered fallback (n=822), n (%)	303 (36.9)
Successfully processed input (n=822), n (%)	519 (63.1)
Correctly processed inputs (n=519), n (%)	359 (69.2)
Inputs with errors (n=519), n (%)	160 (30.8)

Feedback from iteration 3 (formal usability testing) was invaluable (Table 3). According to user feedback, all testers (9/9, 100%) agreed that the automatic generation of family trees saved time and was very intuitive. More than half of the testers (7/9, 77.8%) found the dialogue interface to be user-friendly and easy to use. During the testing process, testers also provided some suggestions, such as the need to ensure the accuracy of the results, the desire for manual editing of family trees, and the hope for features like exporting family trees in PDF format.

**Table 3.** Overview of tester feedback on key features in usability testing.

Problems	Feedback overview (number of people who provided this feedback)
What features work well?	Automatically generates a genealogy chart, which is very intuitive (9) Conversational interface is user-friendly and easy to use (7) Saves a lot of time on manual recording and drawing (9)
What features are not good?	Inability to deal with some complex family relationships (5) Occasionally unable to understand my statement, triggering a fallback (2) The generated family chart cannot be edited directly (8)
Do you think this system meets clinical needs?	Yes, great potential for efficiency (9) Yes, but only if accuracy is guaranteed (7) Yes, it would be better if it could be integrated with an electronic medical record system (5)
What features do you want to add?	Manual editing of family charts (8) Risk assessment summary or “heatmap” (7) Export the family chart as an image or PDF (6)

On the basis of this feedback, we made significant revisions to the system. The IT team embedded the family chart online editing function and the risk heatmap module. We further expanded the database to 200 core information nodes and 1800 user expression examples.

The core challenge revealed by the second and third iterations was the accuracy of genealogy mapping. Therefore, we added a fourth iteration, increasing the number of core information nodes to 245 and nearly doubling the number of recognizable user expression examples to a total of 2256.

After 4 rounds of iterative optimization, the final pilot version of the system achieved significant improvements in various performance indicators. The system successfully processed a total of 2042 pieces of data to generate 218 sets of genealogy charts. The system demonstrated high technical stability, experiencing no software crashes or rendering failures during this testing phase. The data were generated by 37 users, including physicians, nurses, and nonmedical workers.

We selected 50 family pedigrees for analysis. All conversational data and system-generated pedigree maps were cross-checked, and a comparison was made between manually drawn pedigree maps and those generated by AI. The primary verification focused on whether the role and gender of each member node matched. The evaluation of pedigree map construction included checking whether all connections in the diagrams were correct and ensuring the accuracy of parental, spousal, and sibling relationships.

The first step involved verifying node-level information: whether the total number of members matched; identifying whether the role and gender of each member node (eg, grandfather, grandmother, and proband) were consistent; and counting all member nodes in the pedigree map, as well as correctly identified, missing, and extraneously extracted member nodes.

The second step involved verifying relationship-level information: checking whether all connections in the diagram were correct, including parental, spousal, and sibling relationships; and counting all relationships in the pedigree map, as well as correctly identified, missing, and extraneously extracted relationships.

The third step involved verifying clinical attribute-level information: ensuring node matching, attribute keys, and attribute values were consistent for each member, including attributes such as current age, age of onset, disease, age at death, and cause of death.

After the verification, on independent test sets containing complex kinships, the genealogy graph had remarkable precision in node and relationship generation and clinical attribution (Table 4). The length of dialogue in the system varied depending on the complexity of the pedigree, but the content was simple and easy to understand. Upon completing the dialogue, the average time to generate a pedigree was under 3 minutes, which was significantly shorter than the typical 10 to 20 minutes required in clinical settings and greatly contributed to improving the efficiency of clinical genetic counseling.

**Table 4.** Performance evaluation of AI-generated pedigree charts across 3 semantic dimensions.

Dimension and metric	Global counts^a^			Micro-average^e^ (%)	Macro-average^f^ (95% CI)
	TP^b^	FP^c^	FN^d^		
Entities (nodes)					
Precision	—^g^	—	—	99.3	99.23 (98.17-100.00)
Recall	547	4	28	95.1	95.67 (92.96-98.39)
*F*_1_-score (Support:575)^h^				97.2	97.19 (95.39-98.99)
Relations (edges)					
Precision	—	—	—	99.3	99.2 (98.05-100.00)
Recall	1201	9	95	92.7	94.52 (91.00-98.04)
*F*_1_-score (Support:1296)^h^				95.9	96.35 (93.98-98.72)
Attributes (clinical information)					
Precision	—	—	—	98.9	99.32 (98.00-100.00)
Recall	89	1	3	96.7	97.62 (94.95-100.00)
*F*_1_-score (Support:92)^h^				97.8	98.23 (96.24-100.00)

a Global counts are cumulative counts of TP, FP, and FN summed across all 50 samples.

b TP: true positive.

c FP: false positive.

d FN: false negative.

e Micro-average was calculated using global sums: precision=ΣTP/(ΣTP+ΣFP); recall=ΣTP/(ΣTP+ΣFN), representing overall performance at the instance level.

f Macro-average is the arithmetic mean of per-sample metrics. 95% CI calculated as mean±1.96×SE, indicating consistency across samples with different family structures.

g Not applicable.

h Support indicates the total number of ground truth instances (TP+FN).

## Discussion

### Principal Findings

In this study, an intelligent assisted diagnostic decision-making system designed for hereditary gynecological tumors was successfully constructed and preliminarily verified. The final pilot version achieved near-perfect accuracy in generating genealogy nodes and relationships. Although AI functions were initially limited, the final system received positive feedback from test users regarding its innovation, ease of use, and intuitive information presentation. The developed system was an accurate, efficient auxiliary tool. After sufficient clinical validation, it holds promise for integration into routine clinical workflows to optimize genetic counseling services. The entire development process was more challenging than expected, but it turned out that AI tools have the potential to become a powerful complement to traditional genetic counseling models by combining large specialized models with clinical workflows [10,11].

The core strength of this project was its specialization and participatory design. Unlike tools that rely on general large models, the specialized large model we used effectively reduced the risk of generating clinically inaccurate information through knowledge-base construction in the field of gynecological oncology. The participatory design approach used throughout ensured that the functions, interactions, and outputs of the system (eg, the style of the family map) were closely aligned with the actual work habits and needs of clinicians, which is the key to the successful implementation of the technology in clinical practice. It is worth noting that the core information nodes of the database expanded from the initial 100 to the final 245, while the number of user expression examples recognizable by the system increased from 800 to 2256 (the recognition breadth ratio increased from 8.0 to 9.2), reflecting the great efforts we made to improve the AI’s understanding capabilities and the accuracy of genealogy map generation during the development process.

The biggest challenge in the development process, consistent with the literature, was the precise translation of highly unstructured, ambiguous natural language conversations into structured data and knowledge graphs (ie, family maps) that complied with medical genetics norms. Our experience shows that the general capabilities of LLMs alone are not enough to solve this problem. The introduction of the graph-based kinship inference and validation module and the implementation of multiple rounds of data-driven iterative optimization are effective ways to overcome this challenge. The significant increase in the scale of the system’s database intuitively reflects the great efforts made to improve AI understanding capabilities.

The clinical significance of this system is far-reaching. By allowing patients to interact with the system before consultation, family history collection, the longest and most error-prone task in the traditional mode can be efficiently completed. This allows genetic counselors to free valuable clinic time from tedious data entry and mapping work and focus more on in-depth communication with patients, interpreting complex genetic reports, and providing psychological support, thereby greatly improving the quality and accessibility of genetic counseling.

The value of this informatics approach is not limited to accuracy metrics. In routine clinical practice, shifting family history collection, the longest and most error-prone task, to a self-service interaction before the consultation frees counselors from manual charting and lets them spend the saved time on interpretation, risk communication, and psychological support; standardized capture also reduces between-center variation. On the personalized-care side, pairing the gynecology-oncology RAG knowledge base with rule-based risk logic and the graph-based kinship inference module converts unstructured family narratives into individual-specific pedigrees and risk cues, so assessment can follow the patient rather than a generic template. From a population health policy perspective, taking the tool into primary care and community settings would broaden access to pedigree-based screening for high-risk families, supply the data needed for cost-effectiveness analyses, and help shape standardized family history protocols that extend hereditary cancer prevention to the population level.

### Limitations

Although the development of the system involved the participation of multicenter experts across the country, its clinical validation was mainly carried out at a single medical center with a small sample size. Larger-scale, multicenter prospective studies are needed in the future to verify its effectiveness and generalization ability in different clinical settings. During the design phase, despite the full involvement of patient representatives, it would have been better to involve a more diverse patient population in each round of testing. We have initiated a follow-up study to conduct in-depth interviews with patients using the system in a real-world clinical setting, the results of which will be used for the final iteration of the system.

While certain pedigree resources were consulted during the development of the tool, its functionality relies on active user input. The primary focus of this paper is the conversion of natural language into structured pedigree representation; benchmarking against existing pedigree software or other AI tools has not yet been conducted. Currently, most existing pedigree drawing software relies heavily on manual data entry and structured form-filling, lacking the capability to process unstructured, multiturn natural language dialogues. Therefore, conducting a direct, quantitative head-to-head comparison is challenging, as the input modalities are fundamentally different. However, we recognize the importance of this comparison, and such comparisons have been considered for inclusion in subsequent testing.

### Conclusions

Successful implementation of AI tools in health care requires systems that can accurately analyze user inputs and provide correct and reliable outputs. As AI technology evolves to better understand the nuances of written language on a deeper level, patients will be able to fully understand and prepare information through interaction with the intelligent system before meeting with the physician. This not only makes physician-patient communication more efficient and targeted but also makes personalized medicine truly a reality. Tools such as the hereditary gynecological tumor assistance system are expected to become efficient tools for genetic counselors.

## Abbreviations

LLM: large language model
RAG: retrieval-augmented generation
